# Mismatch or allostatic load? Timing of life adversity differentially shapes gray matter volume and anxious temperament

**DOI:** 10.1093/scan/nsv137

**Published:** 2015-11-13

**Authors:** Manuel Kuhn, Robert Scharfenort, Dirk Schümann, Miriam A. Schiele, Anna L. Münsterkötter, Jürgen Deckert, Katharina Domschke, Jan Haaker, Raffael Kalisch, Paul Pauli, Andreas Reif, Marcel Romanos, Peter Zwanzger, Tina B. Lonsdorf

**Affiliations:** ^1^Department of Systems Neuroscience, University Hospital Hamburg-Eppendorf, Hamburg, Germany,; ^2^Department of Psychiatry, Psychosomatics, and Psychotherapy and; ^3^Department of Psychology I, Biological Psychology, Clinical Psychology und Psychotherapy, University of Würzburg, Würzburg, Germany,; ^4^Department of Psychiatry and Psychotherapy, University Hospital Münster, Germany,; ^5^Karolinska Institutet, Department of Clinical Neuroscience, Karolinska Institutet, Stockholm, Sweden,; ^6^Neuroimaging Center (NIC), Focus Program Translational Neuroscience, Johannes Gutenberg University Medical Center Mainz, Germany,; ^7^University Hospital Johann Wolfgang Goethe-University, Department of Psychiatry, Psychosomatics and Psychotherapy, Frankfurt, Germany,; ^8^Department of Child and Adolescent Psychiatry, Psychosomatics and Psychotherapy, University Clinic of Wuerzburg, Wuerzburg, Germany,; ^9^Department of Psychiatry, Ludwig Maximilians University Munich, Germany, and; ^10^kbo Inn Salzach Hospital Wasserburg, Germany

**Keywords:** VBM, childhood maltreatment, adversity, stressful life events, mismatch, allostatic load

## Abstract

Traditionally, adversity was defined as the accumulation of environmental events (*allostatic load*). Recently however, a mismatch between the early and the later (adult) environment (*mismatch*) has been hypothesized to be critical for disease development, a hypothesis that has not yet been tested explicitly in humans. We explored the impact of timing of life adversity (childhood and past year) on anxiety and depression levels (*N* = 833) and brain morphology (*N* = 129). Both remote (childhood) and proximal (recent) adversities were differentially mirrored in morphometric changes in areas critically involved in emotional processing (i.e. amygdala/hippocampus, dorsal anterior cingulate cortex, respectively). The effect of adversity on affect acted in an additive way with no evidence for interactions (mismatch). Structural equation modeling demonstrated a direct effect of adversity on morphometric estimates and anxiety/depression without evidence of brain morphology functioning as a mediator. Our results highlight that adversity manifests as pronounced changes in brain morphometric and affective temperament even though these seem to represent distinct mechanistic pathways. A major goal of future studies should be to define critical time periods for the impact of adversity and strategies for intervening to prevent or reverse the effects of adverse childhood life experiences.

## Introduction

Adversity, in particular early in life, is a major risk factor for the development and relapse of affective psychopathology in adulthood (Ventura et al., 1989; Heim and Nemeroff, 2001; Paykel, 2003; Gilbert et al., 2009, 2014; Norman et al., 2012) and is not only associated with increased morbidity but also increased mortality (McLaughlin and Hatzenbuehler, 2009).

There is abundant evidence that adversity can result in enduring behavioral changes while the (neurobiological) mechanisms mediating the associations between stressful life events and affective psychopathology are not well studied to date. Rodent work has linked adversity to increased anxiety-related behavior as well as to structural alterations in limbic (i.e. hippocampus, amygdala) and cortical (prefrontal cortex, PFC) areas (Lupien *et al.*, 2009; Davidson and McEwen, 2012). In support of this, human neuroimaging studies have revealed corresponding morphological changes following childhood and recent (negative) life stress. Thereby experience-dependent neuroplastic changes have been described in key stress- and emotion-related regions such as frontal areas [medial PFC, anterior cingulate cortex (ACC)] as well as the amygdala and the (para-) hippocampus (Conrad, 2008; Tottenham and Sheridan, 2009; Van Harmelen *et al.*, 2010; Papagni *et al.*, 2011; Ansell *et al.*, 2012; Davidson and McEwen, 2012; Teicher *et al.*, 2012; Opel *et al.*, 2014, 2015). Of note, these changes have been shown to occur within weeks or month following an event (Ansell *et al.*, 2012). In sum, morphometric changes in emotion-related circuits developing in the aftermath of adversity are likely to play a pivotal role in governing individual differences in resilience and vulnerability to future adversity.

These previous studies however are limited in serval ways. First, most studies have been conducted in patients samples hampering an unequivocal attribution of results to adversity as opposed to the disorder itself (Lim *et al.*, 2014). Second, very few studies have simultaneously investigated the impact of both childhood or recent life stress on brain morphology in healthy humans (Bremner, 2002; Cohen *et al.*, 2006; Ganzel *et al.*, 2008; Rao *et al.*, 2010; Ansell *et al.*, 2012; Dannlowski *et al.*, 2012). Third, the possibility of sensitive periods for differential behavioral and neural effects of adversity remains unstudied.

Traditionally, in the framework of the ‘*allostatic load hypothesis*’ (McEwen, 2003), adversity is defined as the accumulation of environmental events. Commonly, a composite score is generated as the sum of stressful events over the lifetime or during specific age periods such as early childhood or recent past. Recently, an alternative view, the (stress coping) ‘*mismatch hypothesis*’ (Schmidt, 2011; Homberg, 2012; Nederhof and Schmidt, 2012) has drawn a lot of attention. According to this hypothesis, a mismatch between the early and the later (adult) environment is critical for disease development. It is assumed that early environmental effects induce adaptive neurobiological and behavioral changes and establish (coping) strategies in the organism that serve preparation of the organism for a life in this environment (‘match’). However, under different environmental conditions (‘mismatch’), these changes may turn out to be rather maladaptive. Such an effect of environmental mismatch is by now well established for metabolic or cardiovascular diseases (Gluckman *et al.*, 2007), while the mismatch concept has not yet been integrated in psychiatric or psychological research in humans. Recently, rodent studies have started to incorporate the mismatch approach of anxiety and first results are promising (Nederhof *et al.*, 2014; Santarelli *et al.*, 2014; Bodden *et al.*, 2015), albeit not yet comprehensive. Appreciation of a more fine-grained differentiation between the presence or absence of stressful life events during multiple developmental periods (‘mismatch hypothesis’) and their impact on anxiety-related behavior in adulthood is thus eagerly awaited and can be not only expected to provide important new insights into the effects of stress on anxiety-related behavior but might also shed light on currently discrepant research findings.

Our study in healthy young participants expands upon prior research in investigating the role of adversity on trait anxiety, depression and brain morphology in key areas of a network implicated in stress and emotion while considering different age periods. Thereby, we expect recent and remote (i.e. childhood) adversity to exert a differential impact on brain morphology, while a congeneric effect on anxiety and depression levels, (partly) mediated by brain morphology, is expected. A deeper understanding on how and when adversity exerts an impact on brain structure, function and personality traits may help promoting and informing the development of targeted treatment or prevention programs.

## Methods and materials

### Participants

Valid data from in total 1158 participants, which were part of a larger ongoing data collection within the framework of a collaborative research center (SFB/TRR 58), were included in the study. All participants were screened to be free from psychiatric disorders by the M.I.N.I. diagnostic interview (Sheehan *et al.*, 1998) prior to inclusion in the study. Data were collected at three different sites (Universities of Münster, Würzburg and Hamburg, Germany). Participants were dichotomized into those with and without a history of childhood maltreatment (CA+and CA–) as well as with and without recent stressful life events (RA+ and RA–) based on the Childhood Trauma questionnaire (Bernstein *et al.*, 2003) as well as the list of threatening events (Brugha *et al.*, 1985), respectively.

In addition, structural magnetic resonance data were available from a subset of participants from the Hamburg sample (*N* = 129, Table 1) as well as a larger replication sample (*N* = 327, Table 1). Childhood and recent adversity groups did not differ in sex distribution while age differences emerged between the RA + and RA– groups (VBM sample) and the CA+and CA– sample (questionnaire sample; Table 1). Participants reporting any family history of psychiatric disorders (first or second degree relatives, *N* = 325) were excluded from all primary analyses because previous studies have conceptualized this as a major stressful life event (Newport *et al.*, 2002), leaving *N* = 833 for analyses. Informed consent was acquired from all participants.

**Table 1.** Sociodemographic information for the VBM samples and the questionnaire samples for participants with or without exposure to childhood adversity (CA+ *vs* CA−) and recent adversity (RA+ *vs* RA−)

VBM	CA+^a^	CA−	statistics	RA+	RA−	statistics
				past year		
*N*	32	97		91	32	
age in yrs(s.d.)	25.5(3.6)	24.8(3.2)	*F*(1,127) = 1.06, *P* = 0.30	24.51(3.3)	26.6(3.3)	*F*(1,121) = 9.39, *P* = 0.003, _p_η2 = 0.07
sex (f/m)	20/12	56/41	Pearsons Chi^2 ^= 0.64	42/49	9/23	Pearsons Chi^2 ^= 0.08

VBM(replication)^b^				RA+	RA−	statistics
				past 3 years		

*N*				245	82	
Age in yrs (s.d.)				26.8(5.7)	25.5(4.6)	*F*(1,325) = 4.54, *P* = 0.03, _p_η2 = 0.01
sex (f/m)				70/175	15/67	Pearsons Chi^2 ^= 0.07

questionnaire	CA+^c^	CA−	statistics	RA+	RA−	statistics

*N*	183	650		569	264	
age in yrs(s.d.)	26.39(6.55)	25.36(5.55)	*F*(1,831) = 4.56, *P* = 0.033, _p_η2 = 0.005	25.43(5.70)	25.91(6.00)	*F*(1,833) = 1.02, *P* = 0.27
sex (f/m)	92/91	371/279	Pearsons Chi^2 ^= 0.102	312/257	151/113	Pearsons Chi^2 ^= 0.523

^a^Of the 32 participants reporting childhood maltreatment, 11 reported emotional abuse, 8 reported emotional neglect, 3 reported physical abuse, 17 reported physical neglect and 2 reported sexual abuse.

^b^The replication sample does not represent a completely independent sample from the main study sample, as the main study sample is included here as well. However, as the CTQ and the LTE were only available from the smaller subsample, the main analyses are based on this restricted sample of *N* = 129 while the replication analyses used a different time period to define recent adversity (past 3 years) and a different psychometric instrument (modified Life Calendar).

^c^Of the 183 participants reporting childhood maltreatment, 66 reported emotional abuse, 40 reported emotional neglect, 23 reported physical abuse, 125 reported physical neglect and 23 reported sexual abuse.

### Voxel-based morphometry

High-resolution T1-weighted structural images (1 × 1 × 1 mm) were acquired using a magnetization prepared rapid gradient echo sequence. A 32-channel head coil was used for data acquisition for all subjects. Gray matter (GM) differences were analyzed by using the voxel-based morphometry (VBM) toolbox (VBM8, version 435, www. http://dbm.neuro.uni-jena.de/vbm/) in SPM8 (Statistical Parametric Mapping; Wellcome Department of Imaging Neuroscience, London, UK). Default settings included a ‘non-linear only’ modulation of the GM. The preprocessed images were smoothed with a full-width at half maximum (FWHM) of the Gaussian kernel of 12 mm. Two sample *t*-tests and regression models in SPM8 were set up using age and sex as covariates.

Based on previous work on the impact of adversity on brain morphology (see Introduction), the hippocampus, amygdala and the anterior ACC were chosen a priori as regions of interest (ROIs) and used for small volume correction (SVC) based on anatomically defined masks (probability threshold 0.7; Desikan *et al.*, 2006). Multiple comparisons were controlled for by using family-wise error correction (FWE) at the voxel-level. For additional exploratory whole-brain analyses, an uncorrected (uc) threshold of *P* < 0.001 and a cluster size of *k* ≥ 15 were used.

### Questionnaires

The German version of the *Childhood Trauma Questionnaire* (CTQ) (Wingenfeld *et al.*, 2010) comprises of 28 items with 5-point Likert scales, designed to retrospectively assess negative childhood experiences of five categories (emotional, physical and sexual abuse as well as emotional and physical neglect). For a distribution of these categories in our study samples see Table 1.

The *List of Threatening experiences* (LTE) (Brugha *et al.*, 1985; Brugha and Cragg, 1990) assesses 12 different categories of life events that occurred during the past 12 month.

The *Spielberger Trait Anxiety Scales* (STAI) (Spielberger *et al.*, 1983) is based on a 4-point Likert scale and consists of 20 questions measuring trait anxiety (as a personal characteristic).

The short version of the German *General Depression Scale* (ADS-K) (Hautzinger and Bailer, 1993) was used to assess depressive symptoms during the past week using 15 items on a 4-point Likert scale.

### Statistical analyses

We tested for both categorical and dimensional effects as a categorical classification is relevant from a clinical perspective, the exploration of dimensional associations is essential for research purposes (Insel *et al.*, 2010). Age and sex were used as covariates in all analyses.

For the categorical classification, separate one-way ANOVAs were calculated with STAI or depression as the dependent and presence or absence of adversity during childhood (CA; based on the CTQ) and recent past (RA; based on the LTE) as the independent variables. For the LTE, the presence of at least one event during the past 12 month leads to the classification of recently experienced adversity. For the CTQ, maltreatment was considered when the participant score was higher than a cut-off value for at least one CTQ subscale [emotional neglect (cut-off: 15), emotional abuse (cut-off: 10), physical neglect (cut-off: 8), physical abuse (cut-off: 8), sexual abuse (cut-off: 8)]. Greenhouse-Geisser corrected degrees of freedom were used when appropriate and an α-level of *P* < 0.05 was considered significant.

In addition, a corresponding analysis was performed with mismatch group as the dependent variable [four groups differing by presence or absence of early and recent adversity, (CA+/RA+; CA+, RA–, CA–, RA+; CA–, RA–)]. This analyses employs a different grouping of participants, as it implies that individuals with exposure to early adversity (and recent adversity) are not one homogenous group but in fact different subgroups.

For dimensional variables, multiple stepwise regression analyses predicted continuous scores of trait anxiety and depression from continuous CTQ and LTE sum scores as well as their product term (i.e. interaction) as well as sex and age. All dependent variables were centered to the mean. In addition, AMOS (Version 22) was used to construct a structural equation model testing the direct effects of childhood and recent adversity on anxious temperament and depression as well as a mediation by volumetric estimates in the ROIs (as indicated by beta-extraction from ROI peak voxels). Starting from a saturated model, backward selection of non-significant paths was performed. In other words, all possible connections were allowed in the initial model and non-significant path were removed for the final model. Level of significance was set at *P* <0.05, two-sided model fit was assessed using root mean square error of approximation (RMSEA) by Browne and Cudeck (1992). Reported regression coefficients reflect standardized betas.

Of note, dimensional CTQ and LTE sum scores did not correlate significantly, *r*(Spearman) = 0.04, *P* = 0.31 while categorical classifications revealed that participant classified as exposed or not exposed during both childhood and recent past were slightly overrepresented (Chi^2^^ ^= 0.012; recent and childhood adversity: *N* = 141, *N*_expected_ = 127; only recent adversity: *N* = 431, *N*_expected_ = 445; only childhood adversity: *N* = 45, *N*_expected_ = 59; no adversity at all: *N* = 221, *N*_expected_ = 207).

## Results

### Childhood and recent adversity and self-reported anxiety and depression

#### Categorical classification

**Fig. 1.**
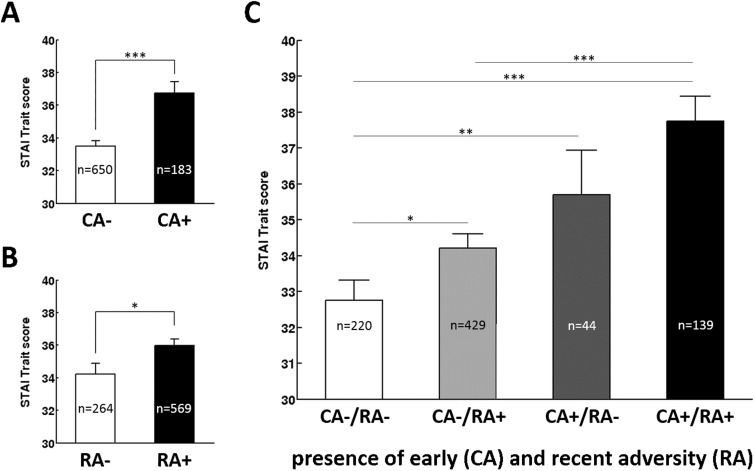
Anxiety (STAI-Trait) scores for participants different by exposure to childhood (A) and recent (B) adversity as well as for the mismatch groups (C; presence or absence of adversity during childhood and /or recent past). Error bars represent SEM. Asterisk indicate statistical significance, ***P < 0.001; **P < 0.01; *P < 0.05 derived from an ANOVA contrasting all four groups.

**Fig. 2.**
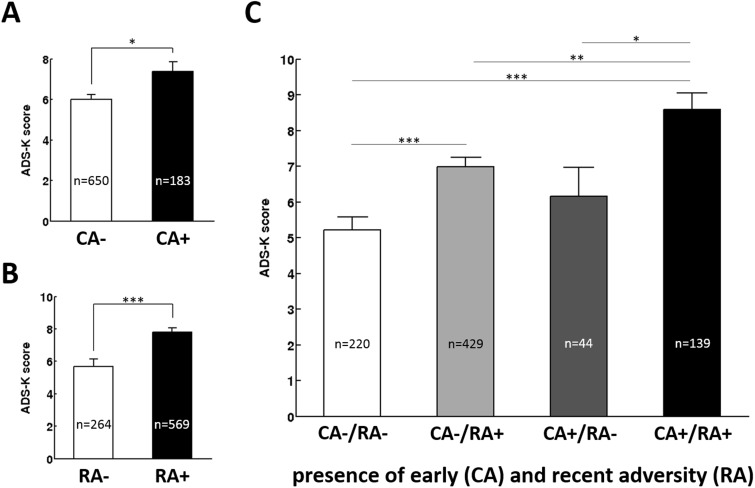
Depression (ADS-K) scores for participants different by exposure to childhood (A) and recent (B) adversity as well as for the mismatch groups (C; presence or absence of adversity during childhood and or /recent past). Error bars represent SEM. Asterisk indicate statistical significance, ***P < 0.001; **P < 0.01; *P < 0.05 derived from an ANOVA contrasting all four groups.

Significant main effects demonstrate that trait anxiety and depression scores were affected by childhood adversity [STAI: *F*(1,827) = 16.924, *P* < 0.001, _p_η^2^^ ^= 0.020; ADS-K: *F*(1,826) = 5.875, *P* = 0.016, _p_η^2^^ ^= 0.007, Figures 1A and 2A] as well as recent adversity [STAI: *F*(1,827) = 4.996, *P* = 0.026, _p_η^2^^ ^= 0.006; ADS-K: *F*(1,826) = 16.344, *P* < 0.001, _p_η^2^^ ^= 0.019, Figures 1B and 2B]. Thereby, participants exposed to childhood (CA+) and recent (RA+) adversity reported more anxiety and depression than those without exposure (CA– and RA–, respectively). Furthermore, no interaction between childhood and recent adversity [STAI: *F*(1,827) < 1, *P* = 0.701; ADS-K: *F*(1,826) < 1, *P* = 0.53; Figures 1C and 2C] or a main effect of sex (both *F*’s < 1, both *P*’s > 0.318) were observed. For age however, a main effect was observed for depression, *F*(1,826) = 6.16, *P* = 0.013, _p_η^2^^ ^= 0.007 but not anxiety, *F*(1,827) = 1.940, *P* = 0.164.

Similarly, an additional analysis grouping participants based on a mismatch approach (CA+/RA+; CA+, RA–; CA–, RA+; CA–, RA–) revealed a significant impact of mismatch group on anxiety, *F*(1,827) = 11.088, *P* < 0.001, pη2 = 0.039 (Figure 1A) and depression, *F*(827) = 11.611, *P* < 0.001, pη2 = 0.040 (Figure 2A) in absence of main effects of sex, both *F*’s < 1.940, both *P*’s > 0.318. A main effect of age emerged for depression, *F*(1,826) = 6.161, *P* = 0.013, pη2 = 0.007 but not anxiety, *F*(1,826) = 1.940, *P* = 0.164. For anxiety, pairwise contrasts revealed that both mismatch groups (CA+, RA–; CA–, RA+) did not differ significantly in STAI values (*P* = 0.253). In addition both groups reporting childhood maltreatment did not differ significantly depending on the presence or absence of recent adversity (CA+, RA–, CA+, RA+, *P* = 0.147) while those that did not report adversity during early or recent past (CA–, RA–) scored significantly lower than all other groups (all *P*’s < 0.033). For depression, participants reporting adversity during both early and recent past scored significantly higher than any other group (all *P*’s < 0.001). In addition, participants without exposure to childhood adversity scored significantly higher when exposed to recent life events (*P* < 0.001) than when not exposed to recent events. All other groups did not differ significantly (all *P*’s > 0.298).

#### Dimensional variables

**Fig. 3.**
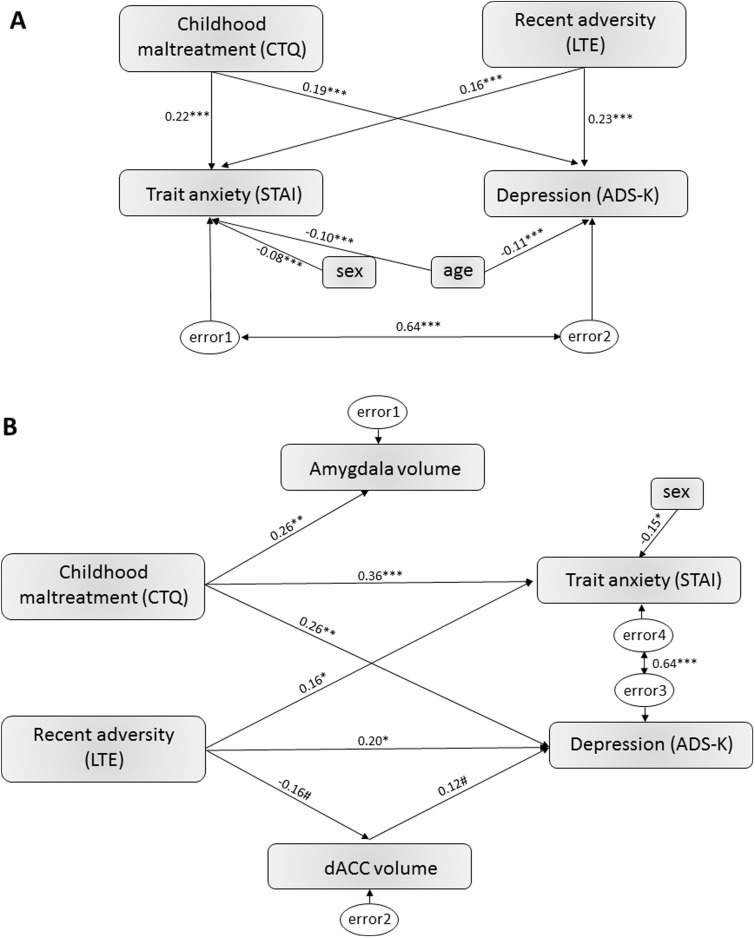
(A) Path model of the relationship between childhood maltreatment and recent adversity on trait anxiety and depression [standardized path coefficients; RMSEA indicated a good model fit (0.017)]. Coefficients for the effect of CTQ and LTE on STAI and ADS-K, respectively, did not differ significantly from each other. (B) Path model of the relationship between childhood maltreatment and recent adversity on trait anxiety and depression including volumetric estimates derived from ROI-based peak voxels as mediating variables [standardized path coefficients; RMSEA indicated a acceptable model fit (0.051)]. Note, that we performed a backward selection of non-significant path starting from a saturated model. Thus paths not included in the figure were non-significant. The indirect (i.e. mediation) effect between recent adversity (LTE) and depression (ADS-K) was not statistically significant (standardized path coefficient: −0.019, 95%CI:−0.067 to 0.001, *P* = 0.055). Asterisks indicate statistical significance with ***P < 0.001, **P < 0.01, *P < 0.05 and #*P* < 0.1.

Multiple stepwise regression analyses were run to predict trait anxiety and depression scores from CTQ and LTE scores as well as their product term (i.e. interaction effect) including age and sex as covariates. A model including childhood adversity, recent adversity, their product term (childhood*recent adversity) and age (only for depression) predicted both trait anxiety, *F*(2,830) = 39.643, *P* < 0.001, R^2^^ ^= 0.295, as well as depression scores, *F*(2,828) = 34.865, *P* < 0.001, R^2^^ ^= 0.335. Both higher childhood and higher recent adversity scores predicted significantly higher trait anxiety scores and depression (all *P*’s < 0.001), supporting a cumulative effect of adversity, while higher age predicted lower depression levels (*P* = 0.003). The interaction term was not significant in either model. A path model for these effects, allowing for more than one dependent measure yielded comparable results which are presented in Figure 3 and were robust to the inclusion of age and sex (not shown).

Additional exploratory stepwise multiple regression analyses showed that higher trait anxiety and depression was significantly predicted (all *P*’s ≤ 0.001) by higher scores of the CTQ subscales *emotional neglect* and *emotional abuse*, [anxiety: *F*(5,827) = 33.661, *P* < 0.001, R^2^^ ^= 0.411; depression: *F*(5,826) = 24.782, *P* < 0.001, R^2^^ ^= 0.361] despite an effect of recent adversity (*P* < 0.001) and age (both *P*’s ≤ 0.003). For depression, also sexual abuse exerted a significant impact (*P* = 0.040) and for anxiety, an impact of sex was observed (*P* = 0.039)

### Differential effects of childhood and recent adversity on limbic and frontal morphometry

**Fig. 4.**
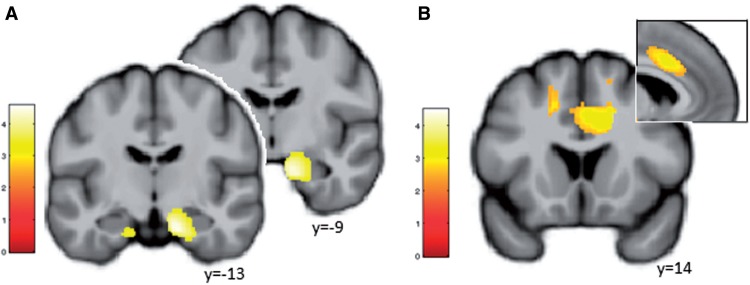
Impact of childhood (A) and recent adversity (B) on volumetric estimates at the amygdala/hippocampus complex as well as the ACC, respectively. Display threshold 0.01uc.

ROI-based analyses revealed significantly larger volumetric estimates in the amygdala/hippocampal complex (both *P*’s < 0.002FWE_SVC,_ Table 2 and Figure 4A) in individuals with childhood adversity (CA+) as compared with those without CA and significantly smaller volumetric estimates in the ACC (*P* = 0.009FWE_SVC,_ Figure 4B) in individuals with exposure to recent adversity (RA+) as compared with those without.

**Table 2.** ROI-based results as well as exploratory whole-brain results at *P* < 0.001 and *k* > 15

	Region		*k_P_*_ <0.001_	*x*	*y*	*z*	*Z*	*P*
CA+ > CA−								
ROI-based	amygdala	right	152	16	−9	−18	3.75	0.002^a^
				18	−4	−23	3.52	0.003^a^
				22	−6	−23	3.34	0.006^a^
	hippocampus	right	256	14	−10	−23	4.38	0.001^a^
whole-brain	parahippocampus	right	1506	22	−21	−27	4.36	0.045^b^
	hippocampus	right		14	−12	−24	4.28	0.063^b^
	hippocampus	left	103	−15	−15	−27	3.32	<0.001uc
CA− > CA+								
whole-brain	cuneus	left	476	−20	−90	9	4.38	0.078^b^
	temporal lobe	left	206	−42	−69	−6	3.45	<0.001uc
	superior frontal gyrus	right	82	8	11	54	3.25	<0.001uc
	temporal lobe	right	17	2	−55	3	3.18	<0.001uc
								
RA− > RA+								
ROI-based	ACC	right	13	6	14	34	3.12	0.031^a^
	superior temporal gyrus	left	2279	−52	−58	13	4.32	0.053^b^
whole-brain	middle occipital gyrus	left	519	−48	−72	−12	4.03	<0.001uc
	superior parietal lobe	left	529	−38	−64	49	3.71	<0.001uc
		left	36	−30	−67	15	3.63	<0.001uc
	superior occipital gyurs	right	132	33	−83	45	3.58	<0.001uc
	middle temporal gyrus	right	278	56	−36	−6	3.58	<0.001uc
	postcentral gyrus	right	447	30	−27	40	3.58	<0.001uc
	medial frontal gyrus	left	75	−15	−1	51	3.48	<0.001uc
	ACC	right	58	18	17	34	3.48	<0.001uc
	precuneus	left	277	−3	−43	45	3.39	<0.001uc
	supramarginal gyrus	right	205	70	−30	30	3.34	<0.001uc
RA+ > RA−								
whole-brain	cerebellum	left		−20	−54	−36	3.38	<0.001uc

^a^FWE_SVC_ at a cluster-forming threshold of *P* < 0.001.

^b^FWE_wholebrain._

Importantly, an explorative replication analysis of a larger sample comparing individuals (*N* = 327, Table 1 for details) with and without exposure to recent adversity [during the past 3 years; classification was based on a modified version of the Life Calendar; (Caspi *et al.*, 2003)] also revealed significantly smaller volumes in the bilateral ACC in participants with exposure to RA, [*x,y,z* = 2,18,16, k_SVC_ = 40, p_FWE(SVC__)_ = 0.015; *x,y,z =*−2,30,16, k_SVC_ = 81, p_FWE(SVC__)_ = 0.009; data not shown], highlighting the robustness of the findings with respect to psychometric measures (LTE *vs* Life calendar). This strongly indicates a sustained effect of life stress on dACC volume reduction which was further supported by significantly higher trait anxiety and depression in this sample [anxiety: *F*(1,823) = 4.71, *P* = 0.03, _p_η^2^^ ^= 0.006 and depression, *F*(1,823) = 10.46, *P* = 0.001, _p_η^2^^ ^= 0.01].

Additional exploratory whole-brain analyses of the main study sample at *P* < 0.001 revealed significantly larger right parahippocampal [*P* < 0.05_FWE(wholebrain__)_] and right hippocampal [*P* = 0.06_FWE(wholebrain__)_] as well as left hippocampal (*P* < 0.001uc) volumes in individuals with CA+. Larger volumetric estimates were observed in individuals without childhood adversity in the left cuneus, bilateral temporal lobe as well as the right superior temporal gyrus. Individuals that had not experienced recent adversity (RA–) additionally exhibited larger volumes in occipital, parietal and temporal areas while only a small cluster in the cerebellum showed larger volumes in individuals with recent adversity.

Further regression analyses revealed an association between the volume of three clusters of the ACC ROI and a dimensional measure of recent adversity [*x,y,z*, 2,32,22; *z* = 3.15, *k*_(SVC__)_ = 9; p_FWE(SVC__) = _0.029; *x,y,z*, 6,14,39; *z* = 3.13, *k*_(SVC__)_ = 5; p_FWE(SVC__) = _0.031; *x,y,z*, 9,17,34; *z* = 3.12, *k*_(SVC__)_ = 4; p_FWE(SVC__) = _0.032] while a regression between the volume of the amygdala ROI and childhood adversity emerged only on a lower exploratory threshold [*x,y,z*, 18,2,−18; *z* = 2.55, *k*_(uc__)_ = 204; *P*_(uc__) = _0.005].

The sample size for the VBM sample did not allow testing for interactions between childhood and recent adversity (mismatch approach) but an exploratory regression analyses including dimensional measures (to maximize power as compared with categorical variables) of childhood and recent adversity as well as their product term did not yield any evidence for an interaction within our ROIs at a lenient threshold of *P* < 0.001 (uc).

### Exploratory analyses including participants with a positive family history of psychiatric disorders

Including participants with a self-reported family history of psychiatric disorders (*N* = 326) did not change the reported results for categorical analyses substantially, that is both childhood and recent adversity were associated with enhanced STAI and ADS-K scores (all *P*’s < 0.005) and childhood adversity manifested as significantly enhanced volume at the right amygdala (*x,y,z* = 16,−6,−20; T = 3.37, *P* = 0.007SVC_(FWE);_ *k*_(SVC_FWE__)_ = 44) hippocampal [*x,y,z* = 14,−10,−23; T = 3.63, *P* = 0.006SVC_(FWE);_ *k*_(SVC_FWE__)_ = 78] junction while recent adversity manifested as volume reduction in the right dACC [*x,y,z* = 6,11,37; T = 3.49, *P* = 0.012SVC_(FWE);_ *k*_(SVC_FWE__)_ = 175] without the emergence of any additional effects within the ROIs (as compared with the results excluding participants with a positive family history of psychiatric disorders).

For dimensional measures, results were also comparable to those not including participants with a positive family history of psychiatric disorders with a significant prediction of both trait anxiety and depression by childhood and recent adversity (all *P*’s < 0.001). Childhood adversity manifests in volume enhancement of the amygdala/hippocampal junction only on an exploratory statistical threshold [*x,y,z* = 15,3,−20, T = 2.71, *P*(uc) = 0.004] while recent adversity manifested as volume reduction in the right ACC (*x,y,z* = 15,20,37, T = 3.52, *P* < 0.001, *k* = 206) even though the cluster was located just outside our ROI.

Furthermore, inclusion of participants with a positive family history (leading to a total *N* of 173 for VBM analyses) did not provide evidence for an interaction between childhood and recent adversity in our ROIs either.

## Discussion

The present data demonstrate a pronounced impact of life adversity on anxious and depressive temperament as well as brain morphology in key regions implicated in stress and emotion. Importantly, timing of adversity (i.e. during childhood or recent past) was differentially mirrored in morphometric alterations of limbic and prefrontal areas while both resulted in enhanced anxiety and depression as a final common pathway. Thereby however, our data do not provide strong evidence that morphometric changes mechanistically mediate the link between adverse experiences and vulnerability for anxiety and depression.

With respect to anxious and depressive temperament, our data provide little support for a mismatch approach of life adversity (Schmidt, 2011; Homberg, 2012; Nederhof and Schmidt, 2012) but rather seem to support the allostatic load hypothesis. In detail, our data demonstrate that both remote (childhood) and proximal (recent) adversity predict higher anxiety and depression directly in absence of an interaction between recent and childhood adversity, in a merely additive (i.e. cumulative) way. That is, no statistical interaction between recent and remote adversity was observed on behavioral and neural outcome measures, which would be predicted by the mismatch approach. In particular individuals exposed to both recent and remote adversity are informative with respect to both theoretical accounts (mismatch *vs* allostatic load), as the mismatch approach would predict low-risk and low-anxiety levels in this group characterized by environmental match, while the allostatic load hypothesis would predict high-risk and high-anxiety levels. As such, our data rather support the allostatic load hypothesis. It has however to be noted, that both, seemingly contradictory, theories may be valid under different circumstances such as individual (e.g. genetic) sensitivity to adversity (Nederhof *et al.*, 2014). It has to be acknowledged, however, that such an interaction between recent and remote adverstiy may in fact also support the allostatic load hypothesis (dependent on the pattern of the interaction) which may provide even stronger support for the allostatic load hypothesis.

In addition to behavioral effects, we observed amygdala and (para-) hippocampal enlargement in adults exposed to childhood maltreatment, while recent adversity was associated with volume reduction of the dorsal ACC, an area critically implicated in emotional expression and appraisal (Etkin *et al.*, 2011). Larger amygdala volumes have previously been linked to childhood adversity (Davidson and McEwen, 2012) such as later age at adoption from an institution as well as subsequent anxiety and internalizing symptoms (Tottenham *et al.*, 2010) and the continuous exposure to a mother suffering from major depression (Lupien *et al.*, 2009) even though also conflicting results have been reported (Lim *et al.*, 2014). In concert with these previous findings, our data support the idea that (childhood) life stress induces structural changes in the (developing) brain. Davidson and McEwen (2012) recently highlighted increased amygdala volume as well as decreased prefrontal volume as the two most prominent structural findings from the human adversity literature. Similarly, evidence from rodents demonstrates that stress promotes excessive growth of amygdala regions (Davidson and McEwen, 2012). Structural changes in this area, which is critically implicated in cognition and emotion (Ochsner and Gross, 2005; DeRubeis *et al.*, 2008; Kim and Whalen, 2009), have been shown to include dendritic debranching and hypertrophy, cell proliferation and synaptic remodeling (Davidson and McEwen, 2012) as well as epigenetic modifications (McGowan *et al.*, 2009). Of note, the morphological changes observed for the amygdala extend into the (anterior) hippocampus, an area rather consistently associated with atrophy (i.e. volume loss) following stress and life adversity (Vythilingam *et al.*, 2002; Teicher *et al.*, 2012; Lim *et al.*, 2014), also in absence of psychopathology (Opel *et al.*, 2014).

An important factor that may contribute to divergent findings in the literature (i.e. hyper- *vs* hypotrophy) is the age at occurrence of adversity. It has been suggested that early hypertrophy (i.e. enlargement) may occur in response to adversity which might later be followed by premature volume reduction (Tottenham and Sheridan, 2009). However, in our sample, childhood adversity was associated with volume *enlargement* in the central hub of the brains emotion processing circuity (Davis and Whalen, 2001) while recent adversity was associated with volume *reduction* in a dorsal ACC region. The morphological differences associated with childhood and recent adversity resemble functional and structural differences observed between healthy controls and patients suffering from anxiety and stress-related disorders as well as depression (Francati *et al.*, 2007; Stuhrmann *et al.*, 2011). In patients, hyperresponsiveness of the amygdala in concert with frontal hyporeactivity is a key finding as well as structural alterations of limbic (amygdala, hippocampus) and frontal (ACC, medialPFC) areas (Bremner *et al.*, 2008). To follow-up on these findings, longitudinal developmental approaches are required.

Importantly, the associations between adversity and affect as well as brain morphology were observed when using a categorical classification (presence or absence of adversity) as well as using a dimensional approach, even though the association between amygdala volume and childhood adversity did not reach formal significance in the latter. This is important, as it suggests, that a categorical classification, as commonly employed in a clinical context, is in fact useful even though dimensional measures are currently favored for research purposes (Insel *et al.*, 2010; Insel, 2014).

Although our results show pronounced effects of childhood and recent adversity on both affect (trait anxiety and depression) and brain morphology (amygdala and dACC volume, respectively), our data suggest that these represent two distinct pathways as brain volume in either region did not predict levels of anxiety or depression. Thus, in contrast to other reports of small effect size (Gorka *et al.*, 2014), our data suggest that even though life adversity manifest as altered volumetric estimates in key regions of an emotion circuitry, this does not seem to reflect the pathway through which changes in trait affect are mediated, even though there was suggestive, albeit not formally significant, evidence that the effect of recent adversity on depression might be partly mediated via dACC volume

Although our study has several major strength such as the large sample size, the consideration of adversity during different critical phases in life as well as its multimodal approach, there are several limitations to the current findings that warrant discussion. First, different group sizes between CA+and CA– as well as RA+ and RA– need to be acknowledged because unequal group sizes can lead to violations of assumptions of the General Linear Model and thus increase statistical error. We however decided against the use of pairwise matched control groups because the benefits of demographic homogeneity have been shown to outweigh the use of the largest possible control group (Pell *et al.*, 2008) in case of retrospective selection of scans from a preexisting pool. In addition the employment of a high smoothing kernel (12 mm) was chosen to render analyses robust against such violations of normality (Salmond *et al.*, 2002).

Moreover, our study design did not allow any conclusion on causality or temporal sequence of effects as results are correlative in nature. Similarly, life adversity was assessed retrospectively, which might be susceptible to memory biases (Sato and Kawahara, 2011). Furthermore, an additional advancement for future studies may be the acquisition of the exact age of trauma occurrence which is not included in many life events questionnaires. Similarly, the CTQ does not place an upper age limit for ‘childhood’ and thus future studies may profit from mapping the age of trauma occurrence in a more fine-grained way.

Furthermore, the current sample consisted of healthy participants, without any (self-reported) family history of psychiatric disorders or prior or current mental disorders. Thus these individuals can be regarded as a sample of people with inherently low vulnerability to develop mood and anxiety disorders, which may explain diverging findings with respect to the impact of adversity on hippocampal volume. In fact, our sample may be considered a particularly resilient sample, which stands in strong contrast to previous studies primarily performed in high-risk patient samples. As it has been suggested that the applicability of the allostatic load *vs* the mismatch hypothesis may depend on an individual’s ‘sensitivity to plasticity’ (Nederhof and Schmidt, 2012), results may in fact turn out differently in high risk or mixed samples. Hence, future studies using longitudinal approaches should explicitly include participants with high vulnerability.

In line with previous findings, our results highlight that morphometric changes, in particular in the hippocampus/amygdala complex as well as the anterior cingulate cortex, may represent a mechanism through which adversity gates stress responsiveness but not individual differences in affective temperament, even though in particular the hippocampus findings are in the opposite direction of what has been reported previously. In particular experimental models of clinical relapse (Vervliet *et al.*, 2013; Haaker *et al.*, 2014) might prove useful in unraveling the mechanisms through which adversity promotes psychopathology in concert with resilience promoting factors (see earlier).

Our data show that life history has a pronounced effect on the behavioral profile in adulthood. Future studies should thus explicitly employ longitudinal designs and systematically target possible buffering factors such as social support (Luby *et al.*, 2012), cognitive-behavioral therapy, coping strategies and positive life events as well as the possible reversibility of structural changes following intervention programs to promote such positive outcomes. A major goal of future studies should be to define critical time periods and mediating factors for the impact of adversity on affect and strategies for intervening to prevent or reverse the effects of adverse childhood life experiences. Although prevention is clearly the preferable route, some degree of reversal of psychopathology and pathophysiology caused by childhood life adversity appears to be an achievable goal.
